# Eye-tracking training improves the learning and memory of children with learning difficulty

**DOI:** 10.1038/s41598-022-18286-6

**Published:** 2022-08-17

**Authors:** Agnes S. Chan, Tsz-Lok Lee, Sophia L. Sze, Natalie S. Yang, Yvonne M. Y. Han

**Affiliations:** 1 Neuropsychology Laboratory, Department of Psychology, The Chinese University of Hong Kong, Shatin, Hong Kong SAR China; 2 Research Center for Neuropsychological Well-Being, The Chinese University of Hong Kong, Shatin, Hong Kong SAR China; 3 Department of Rehabilitation Sciences, The Hong Kong Polytechnic University, Hung Hom, Kowloon, Hong Kong SAR China

**Keywords:** Learning and memory, Neuroscience, Cognitive neuroscience, Therapeutics

## Abstract

Children who experience difficulty in learning at mainstream schools usually are provided with remediation classes after school to facilitate their learning. The present study aims to evaluate an innovative eye-tracking training as possible alternative remediation. Our previous findings showed that children who received eye-tracking training demonstrated improved attention and inhibitory control, and the present randomized controlled study aims to evaluate if eye-tracking training can also enhance the learning and memory of children. Fifty-three primary school students with learning difficulty (including autism spectrum disorder, attention-deficit/hyperactivity disorder, specific learning disorder, specific language impairment and borderline intellectual functioning) were recruited and randomly assigned to either the Eye-tracking Training group or the after-school remediation class. They were assessed on their learning and memory using the Hong Kong List Learning Test before and after 8-month training. Twenty weekly parallel sessions of training, 50 min per session, were provided to each group. Children who received the eye-tracking training, not those in the control group, showed a significant improvement in memory as measured by the delayed recall. In addition, the Eye-Tracking Training group showed significantly faster learning than the control group. Also, the two groups showed a significant improvement in their reading abilities. In sum, eye-tracking training may be effective training for enhancing the learning and memory of children with learning difficulties.

## Introduction

Some children with learning problems find it harder to learn than their peers, and are referred to as children with special educational needs (SEN). Given the adoption of the Integrated Education system in Hong Kong, students with SEN learn together with their classmates in ordinary primary schools. To support Integrated Education, the ordinary school will provide an after-school program to assist their learning or to supplement the learning they obtained in routine classrooms. One common after-school remediation class is to teach students reading and writing skills. The teaching contents are primarily focused on learning vocabulary, word recognition, syntactic skills, reading comprehension, and writing strategies. For example, students are taught to generate short phrases with adjectives and nouns, use common connectives in composition, identify important content words in a paragraph. The purpose of the present study is to compare the effect of a conventional after-school remediation class with an innovative eye-tracking intervention for primary school students with SEN.

The eye-tracking technique has been developed since the 1950s and involves the use of a device that can measure eye movement and gaze positions objectively. This technique has a long history as a research tool and recently has been applied for training and intervention for improving cognitive functions and emotional problems. The rationale of using an eye-tracking system for cognitive training is that eye movement abnormality has been proposed to be associated with frontal lobe impairment such as disinhibition, working memory problems^1–4^. For example, children with attention-deficit/hyperactivity disorder (ADHD) or frontal lobe lesions demonstrated higher error rates in saccadic control (an index of inhibitory control)^2^. The measures of anti-saccade task were correlated with measures of inhibitory control, attention, working memory and self-monitoring^3^. Also, abnormal eye movement control has commonly seen in patients with brain disorders, including Alzheimer’s disease^5–7^, mild cognitive impairment^8–10^, Parkinson’s disease^11–13^, frontotemporal dementia^14–16^, autism spectrum disorders (ASD)^17–19^, ADHD^20–22^, and specific learning disorder (SpLD)^23–25^. Some emerging studies have provided some support for the efficacy and potential of eye-tracking-based training as a cognitive intervention^26–40^. In particular, studies reported positive effects on improving attention, memory and executive functions in post-stroke patients^29,30^, significant improvement in attention and impulsivity in children with ADHD^26,28^, improvement in reading^31,38^, comprehension^31^, executive function^40^, visual attention and verbal memory^39^ in children with dyslexia, reduction in repetitive behaviors^35^, and improvement in visual sustained attention^33^ and emotion recognition^36^ in children with ASD.

Our research team has also studied the effect of a computerized eye-tracking training program. In one of our studies, 18 children with ADHD were randomly assigned to receive the eye-tracking training or web game playing for 240 minutes within two weeks. It was found that only children who have received eye-tracking training showed significant improvements in saccadic eye movement control with shortened saccade latency and higher accuracy in anti- and pro-saccade tasks, respectively^41^. In contrast, those who played with the web game did not show significant changes. Another study revealed significant improvement in various cognitive functions, including inhibitory control, fluency of speech, and flexible thinking in children with ADHD after training^42^. Such positive changes were not observed in the control group that received no such training.

Given the encouraging results of our previous laboratory-based studies, the present study aims to further examine the effects of eye-tracking training as an after-school program to enhance the learning and memory of children with SEN. It also aims to compare the training effects of the eye-tracking training with the conventional after-school remediation classes (i.e., an active control). It is hypothesized that the eye-tracking training will improve learning and memory of the children more than those in the remediation class. As the conventional remediation class teaches Chinese reading and writing skills, it is hypothesized that children receiving conventional training will demonstrate improvement in reading abilities, but may not in learning and memory. Although the eye-tracking training would not teach reading skills as the remediation class, it is hypothesized that the children in the Eye-tracking Training group will show as much improvement in the reading skills as the conventional class given that eye-tracking training has been shown to improve reading abilities in children with SEN^31,37,38^.

## Results

### Baseline performance

The two groups were matched in term of age (*t* = 0.41, *p* = 0.68), gender (*χ*^2^ = 0.25, *p* = 0.61), level of education (*t* = 0.41, *p* = 0.69), estimated level of general intelligence (*t* = 0.59, *p* = 0.56) and diagnostic characteristics (*χ*^2^ = 5.44, *p* = 0.26), they also showed comparable level of ability in the measures of learning, memory and reading abilities, *t* ranges from − 1.49 to 1.37, *p*s from 0.14 to 0.95 (Table 1). The Eye-tracking Training group (*M* = 83%, *SD* = 28%) and the Conventional Training group (*M* = 71%, *SD* = 31%) yielded a similar attendance rate, *t* = − 1.34, *p* = 0.19. No adverse side-effects have been reported in both groups.

**Table 1:** Demographic characteristics and baseline cognitive performance of the Conventional Training and Eye-tracking Training groups.

	Conventional training (n = 24)		Eye-tracking training (n = 26)		*t*/*χ*^2^	*p*
	*M*	*SD*	*M*	*SD*		
Age (years)	9.00	1.04	8.89	0.87	0.41	0.68
Education (years)	3.42	1.02	3.31	0.88	0.41	0.69
Gender (male/female)	19/5		19/7		0.25	0.61
Estimated FSIQ	90.73	11.87	88.78	11.66	0.59	0.56
**Diagnosis**^**a**^					5.44	0.26
With ASD	4		5			
With ADHD	12		6			
With SLI	4		4			
With SpLD	3		7			
With borderline IQ	1		4			
**Learning and memory**						
HKLLT trial 1	3.54	2.34	3.50	1.90	0.07	0.95
HKLLT trial 2	5.67	2.84	4.88	2.64	1.01	0.32
HKLLT trial 3	6.33	3.32	5.46	2.58	1.04	0.30
HKLLT learning slope	1.40	0.93	0.98	1.18	1.37	0.18
HKLLT delayed recall	4.33	3.07	3.50	2.32	1.09	0.28
**Reading ability**						
Chinese word reading score	41.63	27.41	32.88	23.81	1.21	0.23
Chinese passage reading score^b^	125.64	27.37	115.96	34.14	1.07	0.29
Chinese passage reading time^c^	93.96	27.23	111.55	47.80	− 1.49	0.14

*FSIQ* Full-scale intelligence quotient, *ASD* Autism spectrum disorders, *ADHD* Attention-deficit/hyperactivity disorder, *SpLD* Specific learning disorder, *SLI* Specific language impairment, *HKLLT* Hong Kong list learning test.

^a^Participants diagnosed with ASD, ADHD, SpLD or SLI include those with single diagnosis and those with other comorbidities.

^b^n = 22 in Conventional Training group.

^c^n = 21 and 25 in Conventional Training and Eye-tracking Training group respectively.

### Improved learning after eye-tracking training

**Figure 1:**
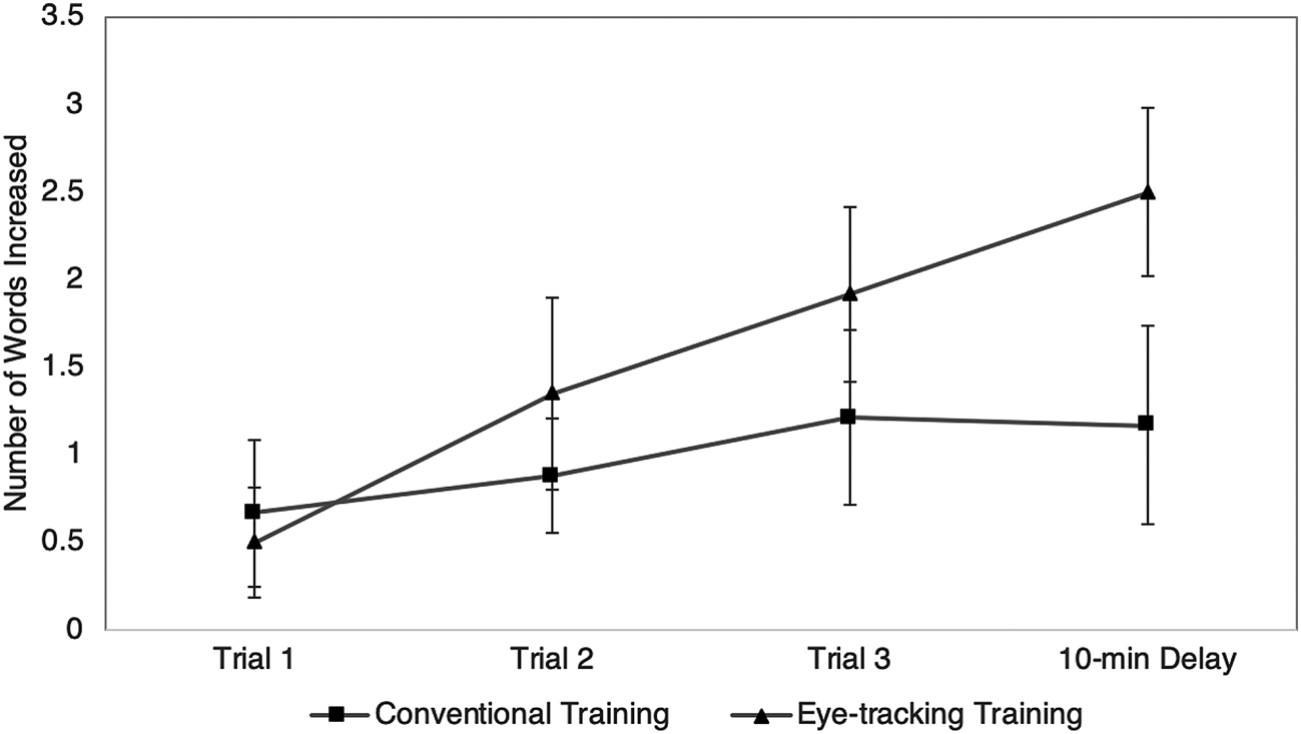
The increase in the number of words recalled across three learning trials and a delayed recall trial of the Hong Kong List Learning Test after training in two groups. The error bar represents one standard error of the mean.

To test out the first hypothesis of whether eye-tracking training will improve learning and memory more than conventional training, a three-way mixed ANOVA was performed with Trials (learning trials 1 to 3 and 10-min delayed recall trial) and Time (pre vs. post) as within-subject factors and Groups (Conventional Training vs. Eye-tracking Training) as between-subject factor. This examines the pre-post change in scores across learning and delayed recall trials between two groups. Although the three-way interaction effect was not significant, *F*(3,46) = 1.29, *p* = 0.29, the partial eta squared (*η*_*p*_^2^ = 0.08) indicated a moderate effect size. The non-significant interaction effect is probably due to our relatively small sample size and hence an inadequate power. Besides, further Trials (learning trials 1 to 3 and 10-min delayed recall trial) by Time (pre vs post) repeated measures ANOVA was performed separately for each training group and the pairwise comparisons with Bonferroni adjustment for *post hoc* analysis was adopted to examine the change in scores after intervention in each group. Table 2 presents the pre-and post-training scores in Learning Trial 1 to 3 of each group, and Fig. 1 showed the difference scores. Pairwise comparison results showed that participants in both training groups did not show any significant change in the first learning trial, *t* = − 1.60 and − 1.61, *p*s = 0.12, a small effect size (*d* = 0.3). In Learning Trial 2, both training groups began to demonstrate a significant increase in the number of words recalled, *t* = − 2.64 and − 2.44, *p* = 0.02, at moderate effect size (*d* = 0.5). At the third learning trial, the Eye-tracking Training group showed an uprising growth in scores and the pre-post change in score became more robust, *t* = − 3.85, *p* = 0.001, approaching a large effect size (*d* = 0.75). The standardized mean difference (SMD) indicating the amount of change in the eye-tracking group relative to the amount of change in the conventional group was computed for each measure to compare the efficacy of two interventions (Table 2). It was revealed that the SMDs for Trial 2 and 3 were positive which suggested a better treatment outcome associated with eye-tracking training. In addition, a two-way mixed ANOVA was performed on the measure of learning slope with Time (pre vs. post) as within-subject factor and Groups (Conventional Training vs. Eye-tracking Training) as between-subject factor. Although the Time by Groups interaction effect was not significant, *F* = 1.68, *p* = 0.20, *η*_*p*_^2^ = 0.03, the *post hoc* pairwise comparisons with Bonferroni adjustment showed that the Eye-tracking Training group demonstrated a significant improvement in learning slope, *t* = − 2.77, *p* = 0.01 (Fig. 1). In contrast, the learning slope of the Conventional Training group was not significant, *t* = − 1.25, *p* = 0.23. The SMD for learning slope was 0.30, which indicated a better outcome for eye-tracking training (Table 2). It suggested that children who have received eye-tracking training were more capable to remember more words after repeated learning.

**Table 2:** Comparison of the number of words recalled at three learning trials and 10-min delayed recall trial, and the learning slope of the Hong Kong List Learning Test (HKLLT) before and after training between two groups.

HKLLT measures	Conventional training (n = 24)		*t*	*p*	*d*	Eye-tracking training (n = 26)		*t*	*p*	*d*	*SMD*
	Pre	Post				Pre	Post				
	*M* (*SD*)	*M* (*SD*)				*M* (*SD*)	*M* (*SD*)				
Trial 1	3.54 (2.34)	4.21 (2.36)	− 1.60	0.12	0.33	3.50 (1.90)	4.00 (1.96)	− 1.61	0.12	0.32	− 0.03
Trial 2	5.67 (2.84)	6.54 (3.18)	− 2.64	0.02*	0.54	4.88 (2.64)	6.23 (2.46)	− 2.44	0.02*	0.48	0.24
Trial 3	6.33 (3.32)	7.54 (3.56)	− 2.42	0.02*	0.49	5.46 (2.58)	7.38 (2.76)	− 3.85	0.001**	0.75	0.37
Learning Slope	1.40 (0.93)	1.67 (0.86)	− 1.25	0.23	0.25	0.98 (1.18)	1.69 (1.17)	− 2.77	0.01*	0.54	0.30
Delayed Recall	4.33 (3.07)	5.50 (3.31)	− 2.05	0.05	0.42	3.50 (2.32)	6.00 (3.19)	− 5.23	< 0.001***	1.03	0.54

SMD = Standardized mean difference, which is computed as [(M_post_experimental − M_pre_experimental)/((SD_post_experimental + SD_pre_experimental)/2)] − [(M_post_control − M_pre_control)/((SD_post_control + SD_pre_control)/2)], where a positive value indicates a better treatment outcome of the experimental group and vice versa.

*p < 0.05; **p < 0.01; ***p < 0.001.

Furthermore, Pearson correlation analyses revealed that children with poorer learning at the baseline demonstrated greater degree of improvement in learning score at later trials (*r* = − 0.60 and − 0.42, *p* = 0.001 and 0.03 for Trial 2 and 3 respectively) but not at the first learning trial (*r* = − 0.38, *p* = 0.06) after eye-tracking training. The degree of improvement in learning score was reflected by the difference score between pre- and post-measurements at each learning trial. In contrast, there was no significant association between baseline score and degree of improvement at later trials for the Conventional Training group (*r* = − 0.07 and − 0.27, *p* = 0.76 and 0.21 for Trial 2 and 3 respectively).

### Improved memory after eye-tracking training

Following the first hypothesis, further analyses were performed to examine if eye-tracking training will improve memory more than conventional training, where memory was assessed by the Delayed Recall Trial. Although the result of mixed ANOVA showed a non-significant Groups by Time interaction effect, *F*(1,48)  = 3.25, *p* = 0.08, it yielded a moderate effect size (*η*_*p*_^2^ = 0.06). Results of *post hoc* pairwise comparisons with Bonferroni adjustment showed that while the Eye-tracking Training group demonstrated a significant improvement in delayed recall, *t* = − 5.23, *p* < 0.001, the Conventional Training group did not show any significant change, *t* = − 2.05, *p* = 0.05, at a small effect size (*d* = 0.42) (Table 2). The extent of change in delayed recall score of the Eye-tracking Training group was at a large effect size (i.e., 1.03), suggesting a great extent of improvement in memory recall after a delay. This is consistent with the positive value of SMD (i.e., 0.54) that eye-tracking training yielded a better treatment outcome than conventional training (Table 2). As shown in Fig. 1, the difference score in the Eye-tracking Training group (*M* = 2.50, *SD* = 2.44) was twice the Conventional Training group (*M* = 1.17, *SD* = 2.79) at Delayed Recall. The results suggest that eye-tracking training, but not the after-school remediation program, improves the memory of children with learning difficulties.

### Improved reading abilities after conventional and eye-tracking training

For the second and the third hypotheses, it is anticipated that the Conventional Training group will show significant improvement in reading abilities, so as the Eye-tracking Training group. Two-way Groups (Conventional Training vs. Eye-tracking Training) by Time (pre vs. post) mixed ANOVAs were performed to test out these two hypotheses. The pre-post performance in the total score of the Chinese Word Reading Test and Chinese Passage Reading Test, and the completion time of Chinese Passage Reading Test were compared between groups. There was no significant interaction effect (*F* ranges from 0.41 to 0.84, *p*s from 0.37 to 0.52), but a significant main effect of Time (*F* ranges from 4.35 to 93.26, *p*s from < 0.001 to 0.04), suggesting that both groups showed improvement after the training. *Post hoc* pairwise comparisons with Bonferroni adjustment were performed to examine the change in reading scores and time after intervention in each group.

**Figure 2:**
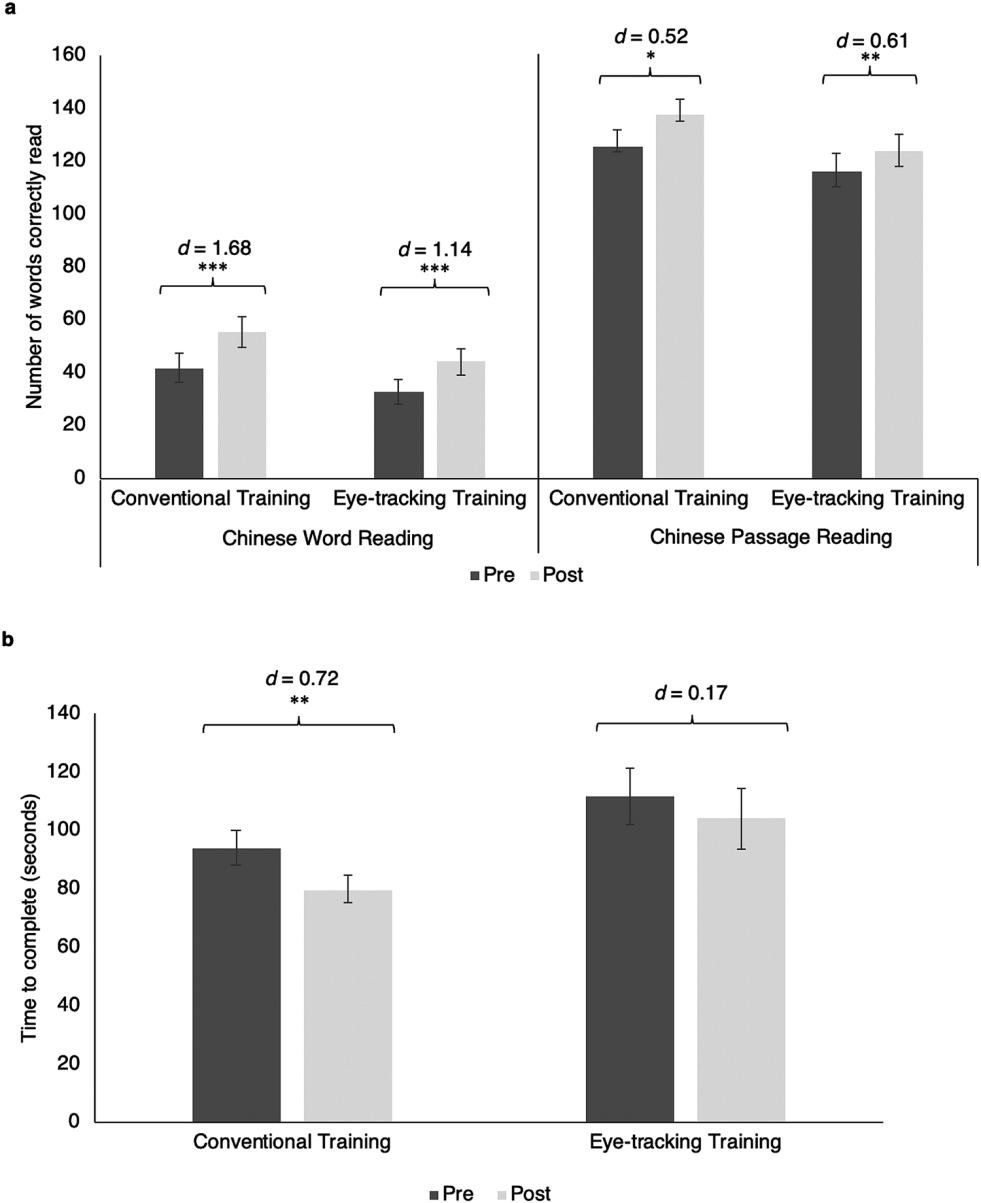
(**a**) The total number of words correctly read in the Chinese Word Reading Test and Chinese Passage Reading Test, and (**b**) the time needed for reading the Chinese passage before and after training. The error bar represents one standard error of the mean. Effect size (*d*) for paired *t*-tests comparing the pre-post difference in each training group is presented. *p < 0.05; **p < 0.01; ***p < 0.001.

Since the students received remediation on their Chinese reading skills in the remediation class, it was not surprising to find that the Conventional Training group showed a significant improvement in the performance of Chinese Word Reading Test (*t* = − 8.20, *p* < 0.001), and Chinese Passage Reading Test (*t* = − 2.44, *p* = 0.02) (Fig. 2a). Interestingly, the Eye-tracking Training group also showed a significant increase in the scores of Chinese Word Reading Test (*t* = − 5.80, *p* < 0.001) and Chinese Passage Reading Test (*t* = − 3.12, *p* = 0.005) after the training. The time to complete the Chinese Passage Reading Test was significantly shorter in the Conventional Training group (*t* = 3.30, *p* = 0.004) but not the Eye-tracking Training group (*t* = 0.85, *p* = 0.40) (Fig. 2b). The extent of improvements in the two reading tests between the two groups was not statistically significant. Therefore, both training groups demonstrated significant improvement in reading abilities.

## Discussion

This study showed that children who received eye-tracking training, not those who attended remediation class, showed improved memory and faster learning. In addition, students with poorer learning scores at the baseline demonstrated a greater degree of improvement in learning score at later trials after eye-tracking training (*r* = − 0.60 and − 0.42, *p* = 0.001 and 0.03 for Trial 2 and 3 respectively). This was not observed in students receiving conventional training. The present result is consistent with a previous study on the eye-tracking training effects on learning and memory. That previous study has applied an eye-tracking linkage attention training program on stroke patients^29^. Forty stroke patients were randomly assigned to receive an eye-tracking training or a conventional computerized cognitive training for 30 sessions, 30 minutes per session. After training, both eye-tracking and conventional cognitive training demonstrated a significant improvement in total learning and a 20-minute delayed recognition of a verbal learning test. These results are consistent with our present findings on the positive effects of eye-tracking training on enhancing verbal learning and memory retrieval at a delay in individuals with brain dysfunctions. However, the extent of improvements was different between Moon et al’s study and the present study. The training-induced increase in delayed recall (*p* < 0.001) in present study was more robust than the improvement in delayed recognition (*p* = 0.042) in Moon et al.’s study. This may be related to the difference in pathological condition and age of participants between two studies (that is adult patients with stroke in Moon et al.’s study and children with learning difficulties in present study). It is understood that memory tested in a recognition format is supposed to be cognitively less effortful than a free recall format because a person is provided with a list of target and non-target words in recognition trial, whereas a person has to recall their memory without any cue provided in a free recall format. Thus, the more robust improvement in delayed free recall in present study is very encouraging and convincing. In addition, the present study is consistent with our previous findings on children with ADHD^40,41^. That is, our previous studies reported better saccadic eye movement control and inhibitory control on children with ADHD after training; the present experiment further showed the positive effects on learning, memory, and reading abilities on children with learning difficulty. Thus, eye-tracking-based training has a positive effect on enhancing cognitive functions including attention, inhibitory control, learning, and memory. More importantly, the improvement in learning and memory after eye-tracking training is supposed to be specifically related to the eye-tracking component of training, rather than simply due to a game or intervention implemented. It is because, based on our previous study^41^, only ADHD children receiving the eye-tracking training but not those engaging in web game playing in the control group demonstrated significant improvement in saccadic latency and accuracy in anti- and pro-saccade tasks. Therefore, it is believed that the positive training effects in present study are possibly due to the eye-tracking component. However, future studies that compare the effect of eye-tracking training and game-based training on various cognitive functions will be warranted.

The present study showed that eye-tracking training was also found to be effective in improving reading skills. This result is consistent with another study that reported significantly faster reading speed and shorter fixation time during reading after 10-minute visual attention training using eye-tracking technology^37^. Similarly, dyslexic children showed reduced reading time and fixation time after 8-week intervention with training component in saccadic control^38^. These suggested training on voluntary eye movement might have benefits on reading skills. Also, another study reported improved reading accuracy, rate and comprehension in dyslexic children after playing action video games via eye-tracking, though such improvements were no different from those playing action games without using eye-tracking^31^. As hypothesized, since eye movement control has been suggested to be related to attention, memory, and reading abilities^43–46^, training on voluntary control of eye movement could enhance memory and reading ability. Since the ability to recall newly formed memory after a delay plays a significant role in students’ everyday school learning, better delayed recall suggests that students can acquire new knowledge and recall what has been learned for later application. So, the present study has provided preliminary support for the efficacy and applicability of the eye-tracking technique in cognitive training as an after-school program for children with learning difficulty in mainstream schools.

Despite the encouraging findings, there are some limitations of the present study. First, the present study has only examined the immediate effect of eye-tracking training, it is unknown if the positive effects could be maintained after the training. Therefore, follow-up studies are warranted to explore the sustainability of training effects. Second, despite the eye-tracking training-specific improvement in memory function, the underlying neural mechanism is still unknown. Therefore, further studies applying neurophysiological measures to explore the neural responses may shed some light on this issue. Third, given the relatively small sample size, further study to validate the present results in different settings and with a large sample is warranted. It is estimated by power analysis that a minimum of 98 children are required in order to achieve a significant three-way interaction effect for the learning and delayed recalled trials, and a minimum of 228 children are required for a significant two-way interaction effect for the learning slope measure. Fourth, the neurodevelopment and education for eight months may have some impact on the improvement in reading skills observed in both groups of training, therefore, it is worthwhile to include a waitlist control group in future studies to explore and compare the extent of developmental and educational influence with the intervention effects.

## Methods

### Participants

A total of 53 students with learning difficulties in grades 2 to 5 at two local mainstream primary schools were recruited through the referral of their school teachers. This sample size was estimated prior to the study through power analysis based on our previous data^42^ applying the eye-tracking training to improve attention and inhibitory control of ADHD children. The mean effect size of pre-post changes after eye-tracking training in various cognitive measures was 0.7. With power at 0.8 and alpha value at 0.05, minimally 19 students per training group were required. If the attrition rate is 30%, then each group requires about 27 students. The age of students ranges from 7 to 10 years (*M* = 8.99; *SD* = 0.96). Forty of them are boys and 13 are girls. According to the record from the Education Bureau, they have been formally diagnosed with at least one type of neurodevelopmental disorders (i.e., ASD, ADHD, SpLD, Specific Language Impairment (SLI), and borderline level of intellectual functioning with IQ score falls in the range of 70 to 79) and/or comorbid with other disorders (Table 1). The diagnosis was formulated by a psychiatrist, a pediatrician, or a clinical psychologist at a general hospital or Child Assessment Centre or by an educational psychologist at school. All of them have a negative history of head injury or epilepsy. This study was conducted according to the guidelines of the World Medical Association Declaration of Helsinki. The research protocol was approved by the Joint Chinese University of Hong Kong—New Territories East Cluster Clinical Research Ethics Committee (Reference number: 2020.501-T).

### Procedure

All students participated voluntarily with written informed consent obtained from their parents before the study. After that, each of them received the baseline assessment to evaluate their level of intelligence, learning, memory, and reading abilities using standardized tests and experimental paradigms individually by trained research assistants. The assessment was conducted at their school.

After the baseline assessment, the participants were randomly assigned into either the Eye-tracking Training group (*n* = 27) or the Conventional Training group (*n* = 26) using the random number generators in SPSS. The two groups were equivalent in age (*t* = 0.20, *p* = 0.85), gender (*χ*^2^ = 0.06, *p* = 0.81), years of education (*t* = 0.35, *p* = 0.73), estimated level of intelligence (*t* = 0.49, *p* = 0.63), diagnostic characteristics (*χ*^2^ = 2.55, *p* = 0.51), learning and memory abilities (*t* ranges from 0.17 to 1.29, *p*s from 0.20 to 0.87), and reading skills (*t* ranges from − 0.56 to 1.24, *p*s from 0.22 to 0.58). Their intelligence quotient (IQ) was estimated by the Matrix Reasoning and Similarities subtests of the Wechsler Intelligence Scale for Children-Fourth Edition (Hong Kong) (WISC-IV(HK))^47^ based on the formula suggested by Aubry and Bourdin^48^. Their learning and memory abilities were measured by the Hong Kong List Learning Test (HKLLT)^49^, and their reading abilities were measured by the Chinese Word Reading Test^50^ and the Chinese Passage Reading Test^51^.

Each group of participants underwent 20 weekly sessions of after-school training, 50 minutes per session, for 8 months. There were two participants from the Conventional Training group and one participant from the Eye-tracking Training group dropped out while training due to the pandemic of COVID-19. The Conventional Training (*n* = 24) and Eye-tracking Training (*n* = 26) groups remained to be matched on their demographic and clinical characteristics, intelligence level, learning, memory and reading abilities, *p*s from 0.14 to 0.95 (Table 1). The details of training are listed below:

#### Eye-tracking training

The eye-tracking training program was developed by our research team and operated by the Pro-talent Association Ltd. in Hong Kong. It is in Phase II of this clinical trial. The training program consisted of six modules and each module had five levels. Participants were required to fixate or trace on a target with their eyes for a certain period and to ignore distractions. Each level of training lasts for 5 or 10 minutes. Participants could choose to continue or quit after each attempt. Whenever the participant obtained a score of 80 or higher, the program would automatically level up.

#### Conventional training

The Conventional Training was composed of paper-and-pencil and computer tasks that focused on training reading and writing skills. The teaching materials were commonly used in Hong Kong. The teaching contents were primarily focused on learning Chinese vocabulary, word recognition, syntactic skills, reading comprehension, and writing strategies. The teaching materials were presented with multimedia and multisensory means to attract and motivate the students to learn, e.g., a training software developed by the Hong Kong Specific Learning Difficulties Research Team of the Hong Kong University was a game-like computerized training teaching word recognition^52^.

At post-training, participants were re-assessed on their intelligence, learning, memory, and reading abilities with the same set of tests as adopted at the baseline. The assessments were conducted by trained research assistants who were blinded to the group assignment.

### Materials

#### Hong Kong list learning test (HKLLT)

The HKLLT^49^ is a verbal memory test that has been developed and widely adopted in local general hospitals and research fields for more than 20 years. It is a reliable and valid assessment of verbal memory abilities associated with different brain pathologies, such as dementia^53^, mild cognitive impairment^54^, schizophrenia^55^, autism^56^. The three learning trials and the 10-minute delayed recall in the HKLLT were administered in present study. Participants have presented verbally a list of 16 two-character Chinese words three times and were required to recall verbally as many words as possible. After 10 minutes, they were asked to recall the words from memory again. The number of words recalled in each of the three learning trials and a learning slope across the learning trials was computed as the measures of learning, and the number of words recalled at the delayed recall trial was used as a measure of memory. The possible range of score is 0 to 16 in each trial, where a higher score means better performance. Besides, the learning slope ranges from − 8 to 8, where a positive learning slope means an increased rate of learning. The verbal learning test was selected to evaluate the learning and memory of the children as it is one of the most common and sensitive tests of memory^57,58^, and the delayed recall task is the most sensitive index of memory decline^59,60^.

#### Chinese word reading test

The Chinese Word Reading Test is a test for literacy adopted from the Hong Kong Test of Specific Learning Difficulties in Reading and Writing for Primary School Students-3rd edition (HKT-P(III))^50^. The HKT-P(III) is a locally developed and validated test that is sensitive to identify children with SpLD with good convergent validity between composite score and subtests (*r* ranges from 0.11 to 0.54) and test-retest reliability of the Chinese Word Reading Test is high (*r* = 0.98). It contained a list of 120 Chinese two-character words and participants were required to read through them one by one until 30 consecutive mistakes were committed. The total number of correctly read words was computed as a measure of reading ability. The score can range from 0 to 120. A higher score indicates better reading ability.

#### Chinese passage reading test

The Chinese short passage, an experimental paradigm, was adopted from the resources released by the Education Bureau^51^, which contained 147 words with the difficulty level equivalent to the primary school level. Participants were asked to read through the Chinese passage accurately and smoothly. The total number of correctly read words and the time taken to read through the passage were computed as measures of reading accuracy and reading speed respectively.

#### Similarities and matrix reasoning subtests

The Similarities and Matrix Reasoning subtests were selected from the WISC-IV(HK)^47^, which is a locally adapted intelligence scale with good reliability (*r*s ≥ 0.9) and validity (correlation among subtests ranges from 0.72 to 0.92). In the Similarities subtest, the participants were required to name the shared characteristics between two verbal concepts. In the Matrix Reasoning subtest, the participants were required to solve visual-spatial problems through analogical reasoning. The scaled scores of the two subtests were used to estimate the full scale IQ of participants based on the formula suggested by Aubry and Bourdin^48^. This formula yields a reliability of 0.88 and a convergent validity of 0.79.

### Data analysis

The between-group difference on demographic and clinical characteristics and cognitive measures was analyzed using independent sample *t*-tests and *chi*-squared tests. To examine if the eye-tracking training will improve learning and memory more than the conventional training, a three-way mixed ANOVA were performed with Trials (learning trials 1 to 3 and 10-min delayed recall trial in HKLLT) and Time (pre vs. post) as within-subject factors and Groups (Eye-tracking Training vs. Conventional Training) as between-subject factor. Further Trials by Time repeated measures ANOVA was performed separately for each training group and the *post hoc* pairwise comparisons with Bonferroni adjustment were conducted to examine the changes in scores across learning and delayed recall trials in each group. To compare the training effects on reading abilities between two groups, two-way Groups (Conventional Training vs. Eye-tracking Training) by Time (pre vs. post) mixed ANOVAs were performed for the reading measures with *post hoc* pairwise comparisons. Partial eta squared (*η*_*p*_^2^), Cohen’s *d*, and SMD were presented to indicate the effect size. The SMD was computed as [(M_post_experimental − M_pre_experimental)/((SD_post_experimental + SD_pre_experimental)/2)] − [(M_post_control − M_pre_control)/((SD_post_control + SD_pre_control)/2)], where a positive value indicates a better treatment outcome of the experimental group and vice versa. All statistical analyses were performed using SPSS 25.0 software.

## Data Availability

The datasets generated during and/or analyzed during the current study are available from the corresponding author on reasonable request.
